# Body shape index versus body mass index as correlates of health risk in young healthy sedentary men

**DOI:** 10.1186/s12967-015-0426-z

**Published:** 2015-02-27

**Authors:** Marzena Malara, Anna Kęska, Joanna Tkaczyk, Grażyna Lutosławska

**Affiliations:** Department of Biochemistry and Biology, Józef Pilsudski University of Physical Education, Box 55, 00-968 Warsaw, Poland

**Keywords:** Body mass index, Body shape index, Insulin, Lipoproteins

## Abstract

Recently a new simply calculated index of body composition -a body shape index (ABSI) has been introduced as an index more reliable than BMI of association between body composition and all-cause mortality. However, until now associations between ABSI and metabolic risk factors have not been evaluated. A total of 114 male university students not engaged in any planned physical activity participated in the present study. Anthropometric measurements (weight, height, waist circumference) were recorded. Body mass index (BMI) was calculated from weight and height, body shape index (ABSI) was calculated from waist circumference, weight, height and BMI. Blood was withdrawn after an overnight fast from the antecubital vein. Triacylglycerols, total cholesterol and HDL-cholesterol levels in plasma were determined using colorimetric methods and Randox commercial kits. Plasma LDL-cholesterol concentrations were calculated according to the Friedewald formula. Circulating insulin was assayed using a standard radioimmunological method with monoclonal antibodies against insulin and BioSource commercial kits. BMI was slightly, but significantly correlated only with circulating TG (r=0.330, p < 0.001) In contrast, ABSI was slightly, but significantly correlated with plasma levels of insulin (r=0.360, p<0.001), TC (r=0.270, p<0.002), LDL-C and non-HDL-C (r=0.300, p<0.001). In participants at the upper quartile of BMI circulating TG was higher (by 50%, p<0.05) than in their counterparts at the lower BMI quartile. Subjects representing the upper quartile of ABSI were characterized by higher plasma levels of insulin, TC, LDL-C and non-HDL in comparison with subjects at the lower ABSI quartile. (by 92 %, 11. %, 29 % and 21 % respectively, p<0.001). ABSI, a new simply calculated index of body fat seems to more accurately depict the variability in circulating insulin and lipoproteins than BMI at least in young, healthy male subjects.

## Background

At present obesity is recognized as the main cause of type 2 diabetes, cardiovascular disease and an important contributing factor in some cancers [1]. In consequence, precise obesity criteria and diagnosis are of special importance in medical practice.

There is a wide range of methods for body fat determination, which are suitable in laboratory practice (BIA, DEXA, CT, and MRI); however, they require costly equipment, which is not always available [2-4]. Much simpler skinfold measurement are time-consuming and have to be performed by experienced technicians [5]. Thus, they are not suitable either for everyday medical practice or in population-based studies. According to WHO recommendations, the body mass index (BMI) calculated from body weight and height and waist circumference (WC) are a valid indicators of fatness and this assumption has been supported by many studies concerning their associations with health risk [6-8]. On the other hand, there are data questioning BMI reliability and indicating that it provides a false diagnosis of body fatness [9,10]. There are also data indicating that regional fat distribution, but not total body fat stores, are related to metabolic disturbances and health risks [11,12]. Furthermore, it has been demonstrated that WHO standards of BMI are not suitable for the evaluation of body fat with respect to ethnicity [13].

Similarly, many doubts exist with respect to associations between BMI and mortality. Assuming that BMI provides reliable information concerning body fatness and taking into account detrimental effects of fat excess on health and mortality, it is not clear why the BMI-mortality relationship is U-shaped, suggesting high mortality in both lean and obese humans [14-16]. Moreover, it is worth noting that in young healthy adults BMI, but also other surrogate indices of fatness (e.g. waist-to-height ratio, body adiposity index) provide poor prognosis of fat mass since they reflect mostly skeletal muscle mass [17,18].

In the literature there are many other simple surrogate indices of body fat such as the waist-to-height ratio (e.g. weight-to-height ratio-WtHR), conicity index - CI, body adiposity index -BAI), however, their validity in respect to BMI is still under debate [19-22]. Recently Krakauer and Krakauer [23] proposed a new simply calculated index of body composition (a body shape index - ABSI) as more reliable than BMI in determination of association between all-cause mortality and body composition. However, data concerning relationship of ABSI with health risk are controversial. ABSI was found to predict resting blood pressure in adolescents more precisely than BMI [24]. On the contrary, ABSI predictive ability was not better than BMI with respect to type 2 diabetes, hypertension and cardiovascular disease in Chinese and Iranian populations [25-27]. The reason for this discrepancy is unknown, however, it may be due to ethnic differences in body fat distribution [28]. In addition, it cannot be excluded that BMI reflecting mostly total body fat differs in its relationship to metabolic variables from ABSI, which encompasses waist circumference, thus at least partially depicts fat distribution [29].

Thus, this study was undertaken and aimed at the evaluation of the relationship between ABSI and BMI and biochemical variables contributing to health risk in sedentary young male adults.

## Methods

### Participants

We studied a sample of 114 male university students recruited through word-of-mouth, and posters displayed at the university and in student dormitories. They were selected from 148 volunteers because they agreed to venous blood withdrawal under fasting conditions. All participants were healthy non-smokers not engaged in planned physical activity and not taking any medication on a regular basis. They were informed about procedures and all provided their written consent. The study protocol was accepted by the local ethics committee at the Jósef Piłsudski University of Physical Education.

### Anthropometric measurements

Body mass was measured to the nearest 0.1 kg and body height to the nearest 0.5 cm using standard medical equipment in subjects wearing light indoor clothing without shoes, jackets and sweaters. Body mass index (BMI) was calculated as body mass (kg) divided by height (m) squared. The subjects’ adiposity was classified according to WHO standards: underweight was defined as BMI < 18.5, normal weight as BMI ≥ 18.5 and <25, overweight as BMI ≥ 25 to BMI <30, and obesity as a BMI ≥30 [30]. Waist circumference (WC) was measured in the midway section between the lower edge of the ribs and the iliac crest with an accuracy of 0.1 cm using non-stretchable tape and values <102 cm were accepted as normal [30]. All measurements were performed twice but in case of divergent results were repeated for the third time. A Body Shape Index (ABSI) was calculated according to Krakauer and Krakauer [23] and the following formula:

$$ \mathrm{ABSI} = \mathrm{W}\mathrm{C}\left(\mathrm{m}\right)/\left[{\mathrm{BMI}}^{2/3} \times \mathrm{height}\ {\left(\mathrm{m}\right)}^{1/2}\right] $$

### Blood tests

Participants were asked to refrain from physical activity for 48 h before blood sampling. They were tested in the morning (8:00–8:30 a.m.) after an overnight fast. Venous blood was collected under aseptic conditions into plastic tubes containing anticoagulant and centrifuged at 4^°^C. Plasma was stored at -70^°^ until analysis.

Glucose was determined using the GOD-PAP method. Circulating glucose was classified according to the International Diabetes Federation with 5.5 mmol/l accepted as the upper level [31]. Triacylglycerols (TG), total cholesterol (TC), and HDL-cholesterol (HDL-C) were assayed colorimetrically. All variables were determined using commercial kits (Randox Laboratories, Great Britain). Coefficients of variation for these analyses did not exceed 5%. The plasma level of LDL-cholesterol (LDL-C) was calculated according to the Friedewald equation [32]. Non-HDL-cholesterol (non-HDL-C) was calculated by subtraction of HDL-C from TC [33]. Concentrations of plasma lipoproteins were classified according to recommendations of the European Atherosclerosis Society and European Guidelines on Cardiovascular Disease Prevention in Clinical Practice (TG <1.7 mmol/l, TC < 4.5 mmol/l, HLD-C >1.0 mmol/l, LDL-C < 2.5 mmol/l, non-HDL < 2.5 mmol/l [34,35]. Plasma insulin was assayed using a standard radioimmunoassay with monoclonal antibodies against insulin and commercial kits (BioSource, Belgium). The sensitivity of the method was 1 μIU/ml, intra and inter-assay coefficients of variation were 6.8% and 9.3%, respectively. All analyses were run in duplicate.

### Statistical analyses

Data are presented as mean ± SD. All variables were checked for normality using the Shapiro-Wilk test. The Pearson correlation coefficients were calculated for logarithmically (e-based) transformed data. Moreover, biochemical variables were interpreted with respect to lower and upper quartile of BMI and ABSI and the Mann–Whitney test was used for data comparison. A p value ≤ 0.05 was considered to be statistically significant. All calculations were carried out using the Statistica v.7 (Statsoft, Illinois, USA).

## Results

Baseline characteristics of the participants are presented in Table 1. According to BMI standards 74.6% of participants were normal weight, 18.4% were overweight, and 7.0% were obese. Only 2.0% of our subjects were characterized by higher than recommended waist circumference. Circulating glucose and triacylglycerols were higher than normal in similar percentages of students (6.1%). Slightly more participants (8.8%) were characterized by lower than normal HDL-cholesterol. On the contrary, higher than normal TC, LDL-C and non-HDL-C were observed in 24.5%, 25.4% and 29.8% of participants, respectively.

**Table 1:** **Anthropometric characteristics and biochemical variables in young healthy men (means ± SD)**

**Variable**	**n = 114**
Age (years)	21.7 ± 1.4
Weight (kg)	77.2 ± 12.2
Height (cm)	180.3 ± 6.7
BMI	23.7 ± 3.3 (25.4)^*^
Waist (cm)	80.7 ± 8.9 (2.0)^*^
ABSI	0.073 ± 0.003
Glucose (mmol/l)	4.8 ± 0.5 (6.1)^^^
Insulin (μIU/ml)	10.5 ± 6.4
TG (mmol/l)	0.9 ± 0.4 (6.1)^^^
TC (mmol/l)	4.5 ± 0.8 (24.5)^^^
HDL-C (mmol/l)	1.4 ± 0.3 (8.8)^^^
LDL-C (mmol/l)	2.7 ± 0.7 (25.4)^^^
Non-HDL (mmol/l)	3.1 ± 0.8 (29.8)^^^

BMI – body mass index; ABSI- a body shape index; TG-triacylglycerols; TC-total cholesterol; HDL-C- HDL- cholesterol; LDL-C – LDL-cholesterol;^*^ percent of participants with overweight and obesity; ^^^percent of participants with disturbed biochemical variables.

BMI was slightly, but significantly correlated only with circulating TG (r = 0.330, p < 0.001). On the contrary, ABSI was slightly, but significantly correlated with plasma levels of insulin (r = 0.360, p < 0.001), TC (r = 0.270, p < 0.002), LDL-C and non-HDL-C (r = 0.300, p < 0.001) (data not shown).

In participants at the upper BMI quartile circulating TG was higher (by 50%, p < 0.05) than in their counterparts at the lower BMI quartile (Table 2). Subjects representing the upper quartile of ABSI were characterized by higher plasma levels of insulin, TC, LDL-C and non-HDL (by 92%, 11. %, 29% and 21%, respectively, p < 0.001) in comparison with subjects at the lower ABSI quartile.

**Table 2:** **Biochemical variables in young healthy men according to lower and upper quartiles of BMI and ABSI**

**Variable**	**Glucose**	**Insulin**	**TG**	**TC**	**HDL-C**	**LDL-C**	**Non-HDL**
**BMI**							
Q1 (20.0 ± 0.9)	4.8 ± 0.6	10.4 ± 7.2	0.8 ± 0.4	4.5 ± 0.9	1.4 ± 0.3	2.7 ± 0.8	3.0 ± 08
Q 4 (28.2 ± 2.8)	5.1 ± 0.4	10.5 ± 4.8	1.2 ± 0.5^a^	4.5 ± 0.8	1.3 ± 0.3	2.7 ± 0.8	3.2 ± 0.8
**ABSI**							
Q1 (0.069 ± 0.001)	4.9 ± 0.5	7.2 ± 2.9	0.9 ± 0.4	4.3 ± 0.8	1.4 ± 0.2	2.4 ± 0.8	2.9 ± 0.8
Q 4 (0.077 ± 0.002)	4.9 ± 0.5	13.8 ± 7.6^b^	0.9 ± 0.4	4.8 ± 0.9	1.3 ± 0.3	3.1 ± 0.8^b^	3.5 ± 0.8^b^

For abbreviations see Table 1; Q_1_^,^ Q_4_ - lower and upper quartile, respectively; ^a^P < 0.05; ^b^P <0.001.

## Discussion

The most important finding of our study concerns ABSI, which is better correlated to changes in circulating TC and insulin than BMI in young sedentary men. It is worth noting that the homogeneity of our participants according to age and sex strengthens our findings. Additionally, taking into account that disturbances in biochemical parameters (insulin, glucose, and lipoproteins) bring about health deteriorations, it could be tentatively postulated that ABSI may be of importance in risk prognosis of type 2 diabetes and/or atherogenesis [36]. On the other hand, it should be stressed that ABSI validity in the prognosis of cardiovascular disease (CVD) is far from being elucidated since Maessen et al. [37] have not found ABSI capable of determining the presence of this disease in middle-aged subjects.

It should be stressed that in some way ABSI agrees with the WHO recommendation concerning waist circumference inclusion into health risk evaluation [30]. Similarly, other authors have suggested that both BMI and WC contribute to the prediction of body adiposity in white men and women [38]. The importance of WC measurements in diagnosis of health risk has been suggested by many authors since it has been postulated that WC provides indirect information about visceral fat accumulation [39,40]. At present it is well documented that visceral fat due to its location and metabolic characteristics contributes to distorted metabolism to a much greater extent than subcutaneous fat [41,42].

However, it is worth noting that mathematical correlations between metabolic variables and surrogate indices of fatness found in our study do not mean a direct cause-effect relationship. On the other hand, marked differences in metabolic profiles of subjects selected according to lower and upper quartiles of ABSI may suggest that ABSI, but not BMI, depicts variability in circulating insulin and lipoproteins in participants of our study mostly characterized by normal body fat according to BMI standards. Thus, it seems feasible that ABSI allows diagnosis of slight metabolic disturbances observed in otherwise healthy subjects [43,44]. In addition, assuming that lower and upper quartiles of BMI varied by 41% (28.2 versus 20.0) and ABSI quartiles differ by 11.6% (0.077 versus 0.069) it seems that even minor changes in ABSI provide information about variability in metabolic risk.

However, more studies are needed to prove this hypothesis, because of limitations of our study: the low number of participants from one social group, living in a big city, and representing one sex and one ethnicity.

## Conclusions

Our study evaluated relationships between two surrogate measures of body composition – body mass index (BMI) and a body shape index (ABSI) with blood biochemical variables which contribute to health risk (glucose and lipoproteins).

Participants classified according to lower and upper quartile of ABSI markedly differ with respect to circulating insulin, total cholesterol, LDL-cholesterol and non-HDL-cholesterol. On the contrary, participants classified according to lower and upper quartile of BMI slightly differ exclusively with respect to circulating triacylglycerols.

Thus, in young and otherwise healthy sedentary men ABSI is a better predictor than BMI of variability in biochemical parameters, which may indicate disturbed metabolic processes.
